# Two-Holder Strategy for Efficient and Selective Synthesis of Lk 1 ssDNA Catenane

**DOI:** 10.3390/molecules23092270

**Published:** 2018-09-05

**Authors:** Qi Li, Jing Li, Yixiao Cui, Sheng Liu, Ran An, Xingguo Liang, Makoto Komiyama

**Affiliations:** 1 College of Food Science and Engineering, Ocean University of China, Nucleic Acids Chemistry and Biotechnology Laboratory, No. 5 Yushan Road, Shinan-qu, Qingdao 266003, China; liqiouc@sina.com (Q.L.); lijouc@ouc.edu.cn (J.L.); cuiyixiaocyx@163.com (Y.C.); liushengac@163.com (S.L.); ar@ouc.edu.cn (R.A.); 2 Laboratory for Marine Drugs and Bioproducts, Qingdao National Laboratory for Marine Science and Technology, Qingdao 266235, China

**Keywords:** DNA nanotechnology, catenane, topologically interlocked rings, linking number, nanostructure

## Abstract

DNA catenanes are characterized by their flexible and dynamic motions and have been regarded as one of the key players in sophisticated DNA-based molecular machines. There, the linking number (Lk) between adjacent interlocked rings is one of the most critical factors, since it governs the feasibility of dynamic motions. However, there has been no established way to synthesize catenanes in which Lk is controlled to a predetermined value. This paper reports a new methodology to selectively synthesize Lk 1 catenanes composed of single-stranded DNA rings, in which these rings can most freely rotate each other due to minimal inter-ring interactions. To the mixture for the synthesis, two holder strands (oligonucleotides of 18–46 nt) were added, and the structure of the quasi-catenane intermediate was interlocked through Watson–Crick base pairings into a favorable conformation for Lk 1 catenation. The length of the complementary part between the two quasi-rings was kept at 10 bp or shorter. Under these steric constraints, two quasi-rings were cyclized with the use of T4 DNA ligase. By this simple procedure, the formation of undesired topoisomers (Lk ≥ 2) was almost completely inhibited, and Lk 1 catenane was selectively prepared in high yield up to 70 mole%. These Lk 1 catenanes have high potentials as dynamic parts for versatile DNA architectures.

## 1. Introduction

Since the pioneering proposal by Seeman [1], a number of DNA nanoarchitectures have been elegantly constructed [2,3,4,5,6]. Among them, DNA catenanes are highly unique and show specific properties which cannot be easily accomplished by other molecular assemblies. Their most significant advantage is their structural flexibility and dynamic features. Their component rings are simply interlocked by a “mechanical bond” without any covalent linkages so that they can mutually rotate each other [7,8,9]. Their topological specificity is also favorable to design highly complicated and sophisticated nanoarchitectures [10,11,12,13,14,15]. In addition, DNA catenanes can be applied to construct versatile molecular machines. For example, Xiu-juan et al. used a strand displacement paradigm to trigger the switchable transition of a DNA catenane across three stations as a DNA rotor [16]. Johann et al. realized the programmed and reversible arrangement of gold nanoparticles by a catenated machine [17]. Some DNA catenanes including specific sequences can transform between two different enzymatic activities and perform various functions as DNAzymes [18] or work as aptamer to detect thrombin with high sensitivity [19]. Furthermore, DNA catenanes can be, when necessary, linked to various DNA nanostructures which are being developed quite rapidly (e.g., DNA origami, DNA boxes, and others) [20,21]. Their relevance to biology and genome science has been also demonstrated [22].

**Scheme 1:**
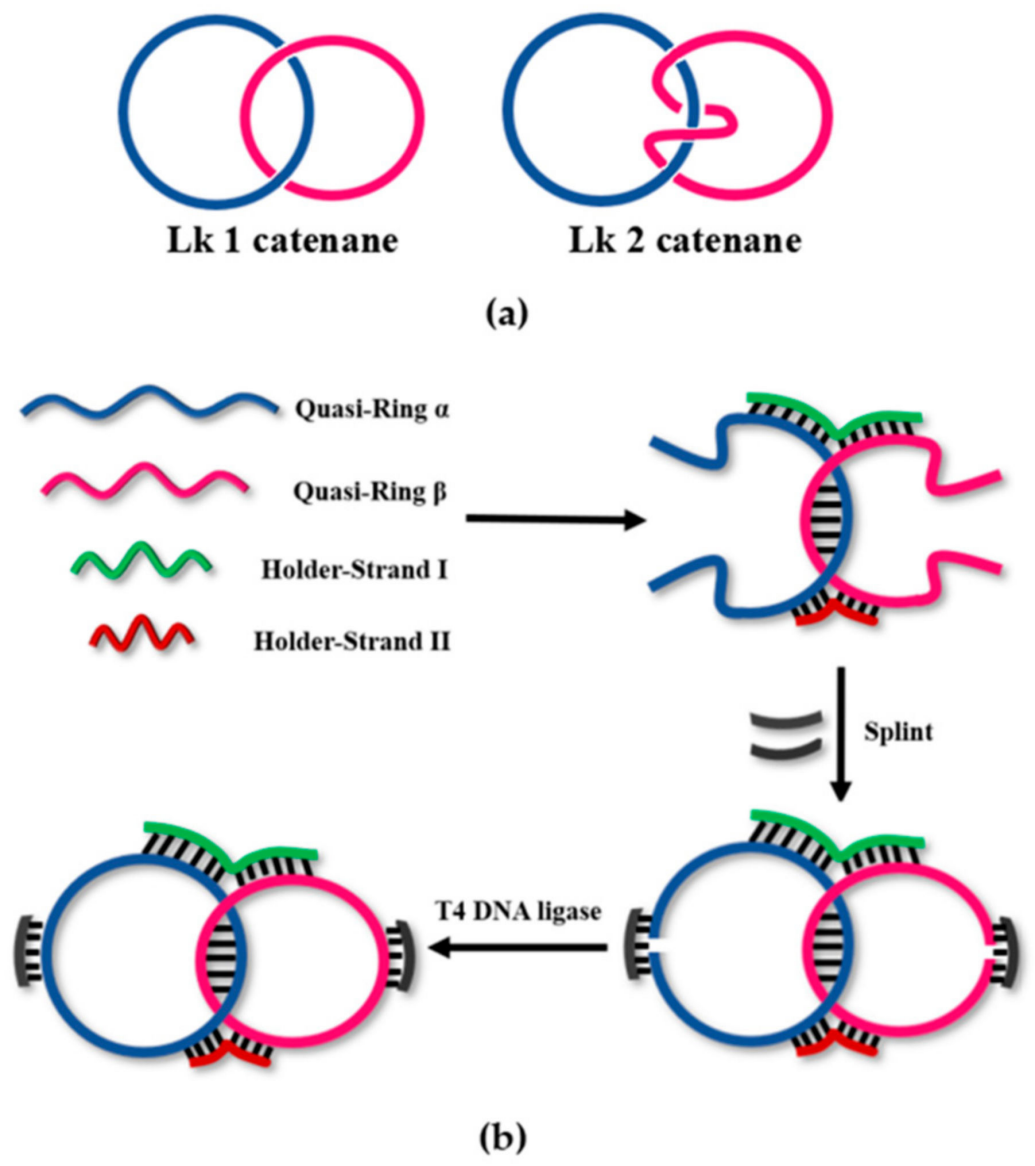
(**a**) The structures of linking number (Lk) 1 and Lk 2 catenanes; (**b**) Two-holder strategy to construct Lk 1 catenane selectively. By using two holder strands (HS_I_, green; HS_II_, red), the twining at the double-stranded portion between two quasi-rings (blue and magenta) is strictly controlled, and the formation of Lk 2 catenane and other topoisomers is suppressed. Of the two holders, HS_I_ mainly draws two quasi-rings close to each other and improves the hybridization efficiency between them. On the other hand, HS_II_ regulates the structure of the X-shaped intermediate, together with HS_I_, and controls the Lk value to 1.

To date, DNA catenanes have been prepared from either single-stranded DNA (ssDNA) or double-stranded DNA. The catenanes of ssDNA have been more widely studied due to larger dynamism and structural flexibility [2,3,4,5,6,23,24,25]. It should be noted that the linking number (Lk) of catenanes (the number of turns intertwining between two interlocked rings) is one of the key factors governing their dynamic properties (see Scheme 1a) [26]. The whole shapes of catenanes are also greatly affected by Lk values. In order to design catenanes of desired functions and integrate them accurately into designated nanostructures, strict regulation of the Lk values in their synthesis is necessary. For many applications, Lk 1 is the most desirable, since then the interactions between the component rings are minimal and the rings can move or rotate most freely [2,3,4,5,6]. If Lk is 2 or larger, the twining of the strands in the rings decreases the freedom of ring rotation (compare the two structures in Scheme 1a). Actually, the linking number for the DNA catenanes shown in Scheme 1a should be −1 or −2 because the DNA duplex is antiparallel. For convenience, in this paper, we used Lk 1, Lk 2, and so on. 

However, selective synthesis of Lk 1 catenane is never an easy task, primarily because of the following two incompatible factors with respect to the length of complementary parts between the quasi-rings [27]. In order to accomplish high yields, it must be sufficiently long (e.g., >15 bp) so that their mutual hybridization in the preparation mixtures (usually at 25–37 °C) is strong enough to facilitate the intermolecular catenation. With the use of a too short complementary part, the hybridization is inefficient and the yield of DNA catenane is low (e.g., <30 mole%). The majority of products are monomeric rings of each quasi-ring. With the use of a too long complementary sequence between quasi-rings, however, Lk 2 catenanes and other higher Lk topoisomers should be more favorably produced than Lk 1 catenanes. Here, the double-helix between the quasi-rings is longer than one helical turn of B-type DNA (10.5 bp), and thus excessive twisting of the strands at the complementary part leads to Lk values larger than 2. In most cases, these two factors are difficult to satisfactorily compromise each other.

In a previous paper [28], the authors developed a new methodology to efficiently prepare catenanes from two ssDNA strands. By adding a holder strand, which is complementary with a part of each of two quasi-rings, the yield of catenation was greatly improved. The holder strand places the quasi-rings close to each other and promotes the catenation. Exactly as designed, the yield of catenane notably increased. With this one-holder method, however, the “trade-off problem” between the selectivity for Lk 1 catenane formation and its high yield could not be completely solved. When the double-stranded portions between the quasi-rings were long enough to attain a high yield, the formation of catenanes of Lk ≥ 2 greatly prevailed. In this paper, we developed a more advanced method in which Lk 1 catenanes are selectively formed in high yields (e.g., 70 mole%). To the reaction mixtures, two holder strands, which form Watson–Crick pairs with both of the quasi-rings at multiple sites, have been added. As a result, the tertiary structures of quasi-catenane intermediates are guided to desirable ones for Lk 1 catenation.

## 2. Results and Discussion

### 2.1. Two-Holder Strategy to Synthesize Lk 1 Catenanes Selectively from Two ssDNA Strands in High Yields 

As illustrated in Scheme 1b, two linear substrate DNA strands, ^L^DNA_α_ and ^L^DNA_β_ (quasi-rings α and β) in blue and magenta, are mixed with a pair of holder strands (HS_I_ in green and HS_II_ in red). ^L^DNA_α_ and ^L^DNA_β_ are complementary with each other in 10 bp, whereas both of HS_I_ and HS_II_ are complementary with some portions of ^L^DNA_α_ and ^L^DNA_β_. As a result, an X-shaped quasi-catenane intermediate involving four Watson–Crick duplex parts is formed (Scheme 1b). Importantly, the double-helix between the quasi-rings α and β is partially rewound due to the formation of four Watson–Crick duplexes (HS_I_/α, HS_I_/β, HS_II_/α, and HS_II_/β), and thus the twining of these two strands is reduced. Apparently, this conformation should favorably lead to Lk 1 catenane, suppressing the production of other catenane isomers (Lk ≥ 2). Then, T4 DNA ligase is added together with ring-closing splints (RS_α_ and RS_β_ in black), which are complementary with the two terminal parts of each of the quasi-rings. Finally, the X-shaped intermediate is covalently fixed by the ligation of the two ends of the quasi-rings, providing Lk 1 catenane selectively. 

**Scheme 2:**
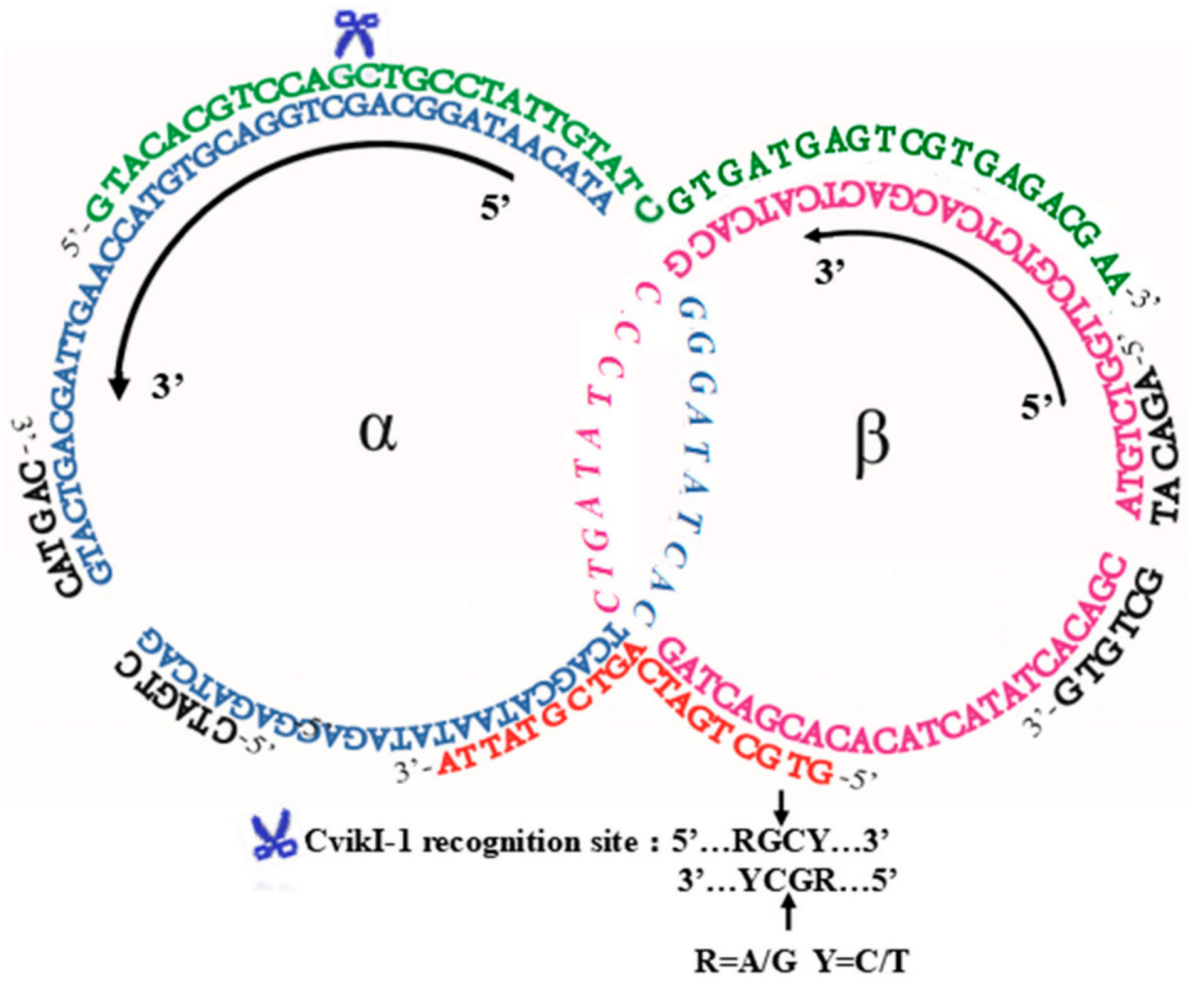
The sequences of two quasi-rings (α (75 nt, blue) and β (63 nt, magenta)), two holder strands (HS_I_ (46 nt, green) and HS_II_ (19 nt, red)), and two ring-closing splints (RS_α_ and RS_β_ (12 nt, black)) which were used for Figure 1. The 10-bp long sequences (italic) in the middle of the quasi-catenane intermediate are the complementary parts between the two rings α and β. The recognition site of restriction enzyme CvikI-1, employed for structural analysis of the catenane product, is placed in the region where HS_I_ is bound to the ring α.

**Figure 1:**
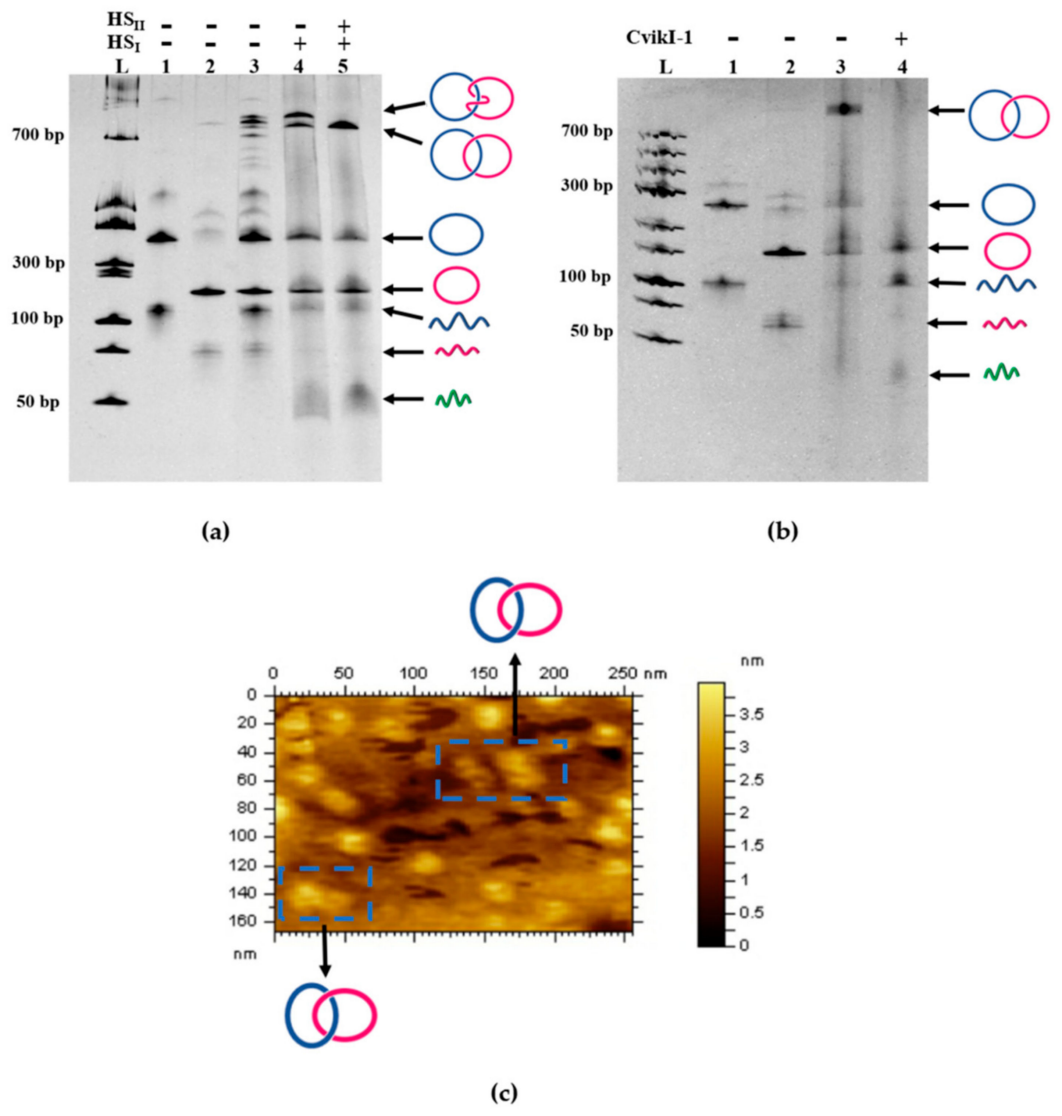
Analysis of a single-stranded DNA (ssDNA) catenane prepared from ^L^DNA_α_ (75 nt) and ^L^DNA_β_ (63 nt) by 14% denaturing polyacrylamide gel electrophoresis (dPAGE). In lane 5 in (**a**), the catenane was fabricated by using two holder strands (46 and 19 nt). [^L^DNA_α_] = [^L^DNA_β_] = [HS_I_] = [HS_II_] = 0.5 μM. After the formation of the quasi-catenane intermediate, T4 DNA ligase was added, together with RS_α_ and RS_β_ (1 μM each). The result of catenation in the presence of only HS_I_ is presented in lane 4, whereas the result in the absence of both HS_I_ and HS_II_ is in lane 3. In lane 4 of (**b**), the products in lane 3 (obtained under the same conditions for lane 5 in (**a**)) were treated with CvikI-1, which digests one site in the α ring (blue) and converts this ring to a linear DNA. In lanes 1 and 2 in both (**a**,**b**), a single ring (^C^DNA_α_ or ^C^DNA_β_), prepared by self-cyclization of ^L^DNA_α_ or ^L^DNA_β_, was analyzed to show its position in the gel. Lane L denotes double strand DNA ladder. (**c**) Atomic force microscopy (AFM) image of the produced catenane.

A typical result of the catenation using two holder strands is presented in Figure 1. As schematically depicted in Scheme 2, two ssDNA strands, ^L^DNA_α_ (75 nt) and ^L^DNA_β_ (63 nt), were converted to the catenane using two holders, HS_I_ (46 nt) and HS_II_ (19 nt). The 25-nt sequence in the 5′-side of HS_I_ is complementary to a part of ^L^DNA_α_ (underlined italics in Table 1), while the other 21 nt of HS_I_ is complementary to ^L^DNA_β_ (italics). Similarly, 10 nt in the 3′-side of HS_II_ are complementary to a part of ^L^DNA_α_ (underlined lowercase), while the other 9 nt are complementary with ^L^DNA_β_ (lowercase). The hybridization part between ^L^DNA_α_ and ^L^DNA_β_ is 10 bp (dotted underline). In order to characterize the ligation product as DNA catenane, the recognition site of restriction enzyme CvikI-1 was designed in the region where HS_I_ binds to the ring α.

As shown in lane 5 of Figure 1a, one strong band assignable to a catenane product was formed (the band near the top of the gel). Apparently, only one topoisomer (Lk 1) was produced by the present catenation (vide infra). The catenation yield was about 70 mole%. The minor products were monomeric rings of ^L^DNA_α_ and ^L^DNA_β_ formed by their self-cyclization (in the middle of the gel). When only one holder strand HS_I_ was used (lane 4), however, two catenanes were formed as the main products (the two bands near the top of the gel) [29,30]. One of them comigrated with the main peak in lane 5, whereas the other migrated more slowly. As shown below, the band of larger mobility in this 14% denaturing polyacrylamide gel electrophoresis (dPAGE) is assignable to Lk 1 catenane, whereas the band of smaller mobility is for Lk 2 catenane. This is probably because the aperture of polyacrylamide gel with high concentration is small, and Lk 1 catenane is more flexible and softer than Lk 2. Therefore, Lk 1 is much easier to pass through the hole of the gel, which results in the larger mobility in 14% dPAGE. In 4% dPAGE, the aperture of the gel is large enough and the integral structure of Lk 2catenane is more compact than Lk 1. So, Lk 2 needs less time to pass through the hole, which results in the larger mobility in the gel [27,29]. In the presence of HS_I_ alone, both catenane isomers were produced in comparable amounts. Moreover, in the absence of both of the holder strands HS_I_ and HS_II_ (lane 3), the yield of the catenation products was much lower, and self-cyclization of ^L^DNA_α_ and ^L^DNA_β_ prevailed. Still more critically, many bands assignable to catenanes were observed in the top of dPAGE. Apparently, the catenation without the two holder strands produced many catenane isomers involving different Lk numbers.

### 2.2. Evidence for Catenane Formation

In order to confirm that the two rings α and β from ^L^DNA_α_ and ^L^DNA_β_ were really interlocked without any covalent linkage, the product in lane 5 of Figure 1a (also in lane 3 of Figure 1b) was digested by the restriction enzyme CvikI-1, which has a cognate scission site in the ring α (but not in the ring β). Exactly as expected, the band for the catenane disappeared (lane 4 in Figure 1b). The ring α as the self-cyclization product was also converted to the linear product. Thus, the interlocking of the rings α and β by a mechanical bond was substantiated. We also treated the catenation products in lane 5 of Figure 1a (lane 3 in Figure 1b) with Exonuclease I, which should remove the linear ssDNA (quasi-rings and linear polymers). It was found that Exonuclease I never digested this product, confirming that it has a cyclic structure and thus no 5′/3′ ends (data not shown). Furthermore, atomic force microscopy (AFM) supported the predicted nanostructure of the catenane (Figure 1c). Each DNA ring has a diameter of approximately 13 nm, which is consistent with the theoretical sizes of the α and β rings.

### 2.3. Verification of Lk 1 Structure of the DNA Catenane Synthesized by the Two-Holder Strategy

**Figure 2:**
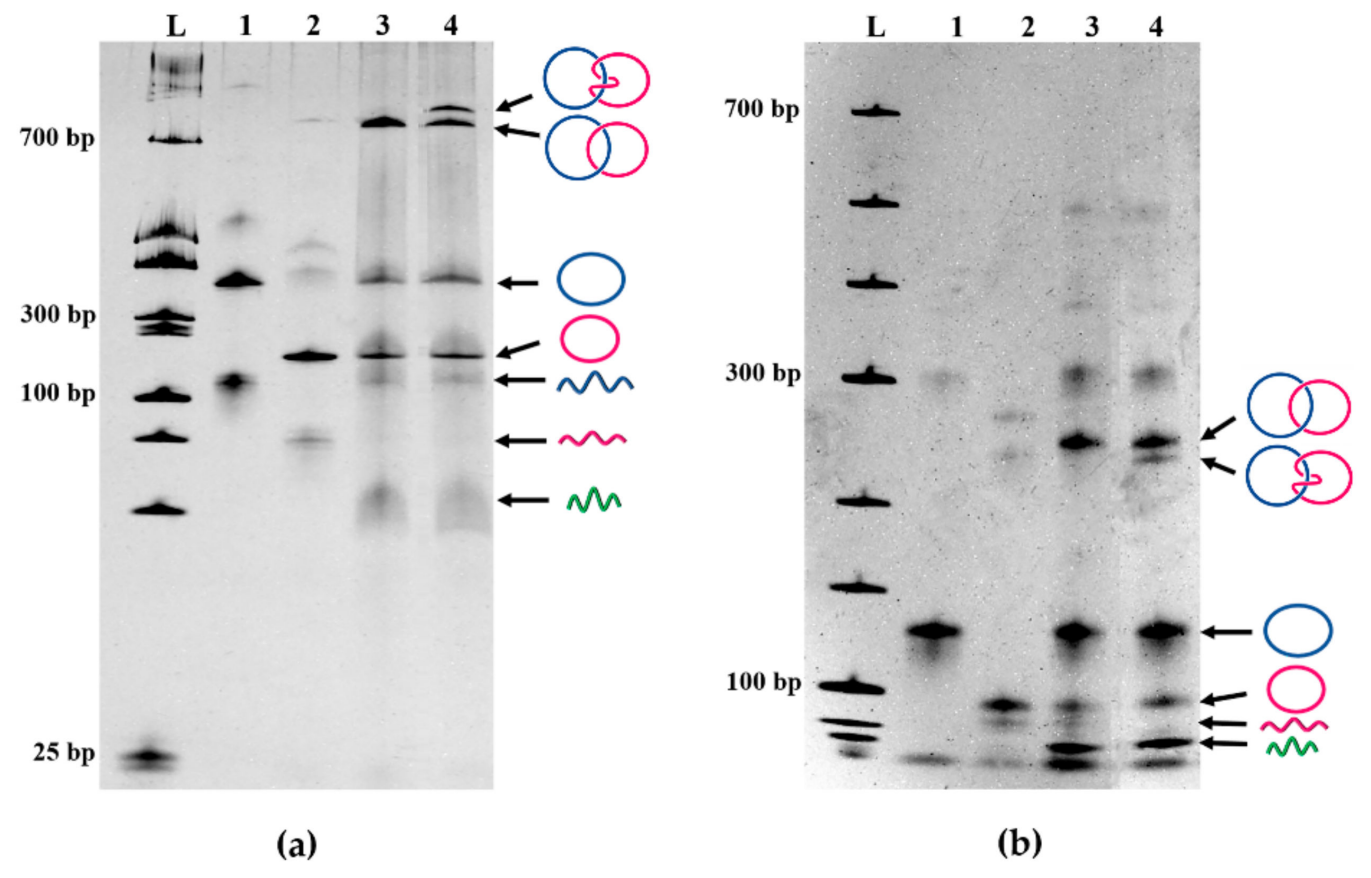
Verification of Lk 1 structure of DNA catenane fabricated by two-holder method. (**a**) Analysis of the catenation products by 14% dPAGE and (**b**) analysis of the same products by 4% dPAGE. For both (**a**,**b**), lane 3 shows the products obtained with the use of both HS_I_ and HS_II_, whereas lane 4 is for the reaction using only HS_I_. In lanes 1 and 2, a single ring, independently prepared from each quasi-ring, is analyzed to show its position in the gel.

In Figure 2a, the products of the α/β catenation using two holder strands (HS_I_ and HS_II_) in lane 3 were compared more in detail with those of the reaction using only one holder strand (HS_I_) in lane 4. The assignment of the two catenane bands in lane 4 has been made in terms of the remarkable dependency of mobility of these two catenanes on the concentration of the polyacrylamide gel used for electrophoresis. According to the literature [27], in the gels of high concentrations (>11%), Lk 1 catenanes show larger mobility than the corresponding Lk 2 catenanes. However, the mobility order is reversed in the gels of low concentrations (4%). As shown in lane 4 of Figure 2a (on a 14% dPAGE), the stronger band (tentatively assigned above to Lk 1 catenane) moved faster than the weaker band assigned to Lk 2. In 4% dPAGE (lane 4 in Figure 2b), however, the stronger band moved more slowly than the weaker band. Undoubtedly, the slower band in lane 4 of Figure 2a is assignable to Lk 2 catenane, and the faster band is assigned to Lk 1 catenane. It is reconfirmed that the Lk 1 catenane has been selectively prepared with the use of both HS_I_ and HS_II_.

### 2.4. Effects of the Lengths of Holder Strands

**Figure 3:**
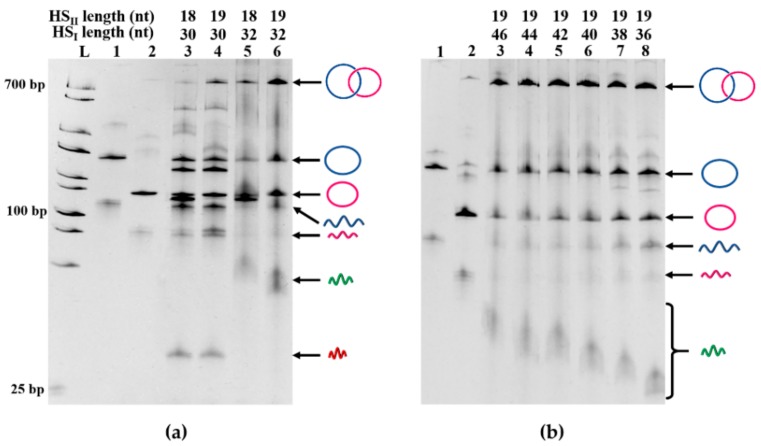
Catenation of ^L^DNA_α_ and ^L^DNA_β_ using two holder strands of various lengths. (**a**) Effect of the length of shorter holder strand HS_II_ on the catenation; (**b**) Effect of the longer holder strand HS_I_ when combined with 19-nt HS_II_.

As long as the holder strands were relatively short, their lengths exerted notable effects on the selectivity for Lk 1 catenane formation and its yield (Figure 3a). In order to obtain Lk 1 catenane efficiently, the lengths of HS_I_ and HS_II_ should be at least 32 nt and 19 nt, respectively. These values correspond to the minimal lengths of DNA duplexes which are required to alter the structures of the double-helixes and form the appropriate X-shaped intermediate for Lk 1 catenation. When the holder strands (both HS_I_ and HS_II_) were sufficiently long, however, the changes in their lengths induced only marginal effects, and Lk 1 catenane was always obtained in high selectivity and high yield (the lengths of HS_I_ and HS_II_ are 36–46 nt and 19 nt, respectively, in Figure 3b). One of the holder strands (HS_I_) primarily promotes the duplex formation between two quasi-rings and facilitates catenation, as clearly evidenced previously [28]. On the other hand, the main role of HS_II_ is to guide the formation of the X-typed intermediate in cooperation with HS_I_ and to control the Lk number of the product to 1.

### 2.5. Successful Preparation of Lk 1 Catenane Even in the Presence of Mismatches between Quasi-Rings

**Figure 4:**
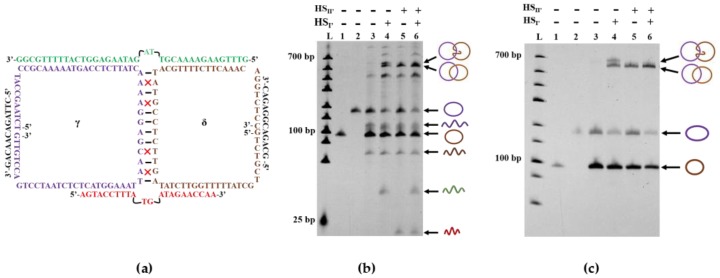
Successful preparation of Lk 1 catenane in the presence of only four consecutive complementary base pairings between two quasi-rings γ and δ. (**a**) Sequences of two quasi-rings γ (72 nt) and δ (59 nt) as well as of two holder strands of HS_I′_ (38 nt) and HS_II′_ (22 nt). There are four mismatches (marked as red crosses) in the 12-bp complementary part between two rings. Two base pairings, in the middle of HS_I′_ and HS_II′_, are kept out of the hybridization region between the holder strand and two rings to minimize the spatial hinderance and make the two rings intertwine more easily; (**b**) dPAGE analysis (14%) of the catenation products. Lane 1, the γ ring; lane 2, the δ ring; lane 3, catenation in the absence of HS_I′_ and HS_II′_; lane 4, catenation in the presence of only HS_I′_; lane 5, catenation in the presence of only HS_II′_; lane 6, catenation in the presence of both HS_I′_ and HS_II′_; (**c**) Treatment of the ligation products in (**b**) with Exonuclease I to remove the linear ssDNA byproducts.

As mentioned previously, another benefit of the holder strands is the improvement of hybridization efficiency between two quasi-rings even when the complementary region between them is short (e.g., 10 bp). In the preparation of DNA catenane using 46-nt HS_I_, at least 21 nt in HS_I_ is complementary to a part of quasi-ring α or β. At the incubation temperature below 37 °C, the corresponding duplexes are almost completely formed. Thus, the hybridization between two quasi-rings transforms the catenation from an intermolecular reaction to an intramolecular one, which results in a high yield of DNA catenane. Consistently, the catenation is very efficient even when there exist many mismatches in the double-helix part between two quasi-rings. For example, the catenane was produced from ^L^DNA_γ_ and ^L^DNA_δ_ in 60 mole% yield, although only 8 bp are complementary between these two quasi-rings, and the consecutive complementary part is only 4 bp (Figure 4a). With the aid of two holders (38-nt HS_I′_ and 22-nt HS_II′_), Lk 1 catenane was successfully obtained (lane 6 in Figure 4b). In the absence of the holders, however, the catenation never occurred (lane 3). Apparently, the X-shaped quasi-catenane intermediate is efficiently formed even under these otherwise unfavorable conditions. The two holders (mainly HS_I′_) assist the close location of two quasi-rings in place of direct hybridization of their complementary parts. Furthermore, they (mainly HS_II′_) also regulate the structure of the X-shaped intermediate for selective formation of Lk 1 catenane. When only HS_II′_ was used without HS_I′_, the selectivity was high, but the yield was considerably lower (lane 5). With the use of only HS_I′_, Lk 2 catenane was produced in a considerable amount (in the upper part of lane 4). These results are completely consistent with the previous arguments regarding the roles of these two holder strands.

In Figure 4c, the products in Figure 4b were treated with Exonuclease I to remove the linear byproducts in the catenation reactions. The selective formation of Lk 1 catenane involving no 5′/3′ ends was still more vividly confirmed.

## 3. Materials and Methods

### 3.1. Materials

DNA oligonucleotides were purchased from Sangon Biological and Biotechnology Co. Ltd. (Shanghai, China) and purified using PAGE before use. The concentration of each oligonucleotide was determined spectroscopically. All other chemicals were commercially obtained.

### 3.2. Preparation of α/β-Catenane Using Two Holder Strands

In a total volume 20 μL of 1× T4 DNA ligase Buffer (40 mM Tris-HCl, 10 mM MgCl_2_, 10 mM DTT, 0.5 mM ATP, pH 7.8 @ 25 °C), ^L^DNA_α_, ^L^DNA_β_, HS_I_, and HS_II_ were mixed. The mixture was heated to 90 °C for 3 min and cooled gradually (0.1 °C/s) to 25 °C. Then, RS_α_ and RS_β_ were added, and the mixture was further incubated for 20 min. The ligation reaction by T4 DNA ligase (5 U) was achieved at 25 °C for 2 h. Finally, the reaction mixture was analyzed on a denaturing polyacrylamide gel (dPAGE). DNA bands were visualized by staining with SYBR Green II and detected by a Universal Hood II Gel Imaging System (Bio-Rad, Hercules, CA, USA). When necessary, the catenanes were purified by gel extraction kit (D2500-01, Omega Bio-Tek, Guangzhou, China) after cutting its band from the gel. The γ/δ-catenane was fabricated in a similar way.

### 3.3. Preparation of Monocyclic DNA

Monocyclic DNA (^C^DNA_α_, ^C^DNA_β_, ^C^DNA_γ_, or ^C^DNA_δ_), as a reference compound for dPAGE, was prepared by mixing 0.5 μM of linear DNA (^L^DNA_α_, ^L^DNA_β_, ^L^DNA_γ_, or ^L^DNA_δ_) and 1 μM of the corresponding ring-closing splint (RS_α_, RS_β_, RS_γ_, or RS_δ_). The mixture was heated at 90 °C for 3 min and gradually cooled to 25 °C. Then, T4 DNA ligase (2.5 U) was added, and the reaction was carried out for 2 h.

### 3.4. Restriction Digestion of α/β-Catenane by CvikI-1

The recognition sequence RGCY of CvikI-1 restriction endonuclease was incorporated within ^C^DNA_α_. The products of DNA catenation described above (17 μL) were mixed with 2 μL of 10× CutSmart reaction Buffer (500 mM CH_3_COOK, 200 mM Tris-CH_3_COOH, 100 mM CH_3_COOMg, 1 mg/mL BSA, pH 7.9 @ 25 °C), followed by adding 1 μL CvikI-1 (5 U/μL).

### 3.5. Denaturing PAGE

All the reaction products (monocyclic DNA, catenanes, and digestion fragments) were analyzed on a series of denaturing polyacrylamide gels (4–14%, acrylamide/bisacrylamide = 29/1 *w*/*w*). To the gel, 19% urea and 11% formamide were added. Unless stated otherwise, the gel electrophoresis was run at 37 °C in 1× TBE (90 mM Tris, 90 mM boric acid, 1 mM EDTA, pH 8.0 @ 25 °C). The staining and imaging were described above.

## 4. Conclusions

In this paper, we have established an efficient methodology to synthesize ssDNA catenanes in which the Lk is strictly regulated to 1. The rings in these catenanes should be able to freely rotate without a significant energy barrier. The key point for the successful synthesis is to add two holder strands to the reaction mixtures. In the resultant X-shaped quasi-catenane intermediate, excess twining of the double-stranded portion, derived from mutually complementary parts of quasi-rings, is avoided, and thus the formation of catenanes of Lk ≥ 2 can be successfully suppressed. Another merit for this approach is that the linking number can be controlled for most sequences, in spite of the GC content, and secondary structures are formed because the two holders are completely complementary to the two strands. The present method can be easily extended to the preparation of catenanes involving multiple rings, in which all the topologies between adjacent rings are regulated to Lk 1. These catenanes should show superb dynamic properties and could be important players in advanced nanoarchitectures. Preparation of these multiple catenanes, as well as their applications to molecular machines, is currently under way in our laboratory.

## Figures and Tables

**Table 1:** Sequences of oligonucleotides used here.

Strand	Sequences (5′–3′)	Length (nt)
**^L^DNA_α_**	p-GACTAGAGCAGATAtaatacgactCACTATAGGG*ATACAATAGGCAGC* *TGGACGTGTAC*CAAGTTAGCAGTCATG	75
**^L^DNA_β_**	p-ATGTCTGG*TTCGTCTCACGACTCATCACG*CCCTATAGTG gatcagcacACATCATATCACAGC	63
**HS_I_**	*GTACACGTCCAGCTGCCTATTGTAT* *CGTGATGAGTCGTGAGACGAA*	46
**HS_II_**	gtgctgatcagtcgtatta	19
**RS_α_**	CTAGTCCATGAC	12
**RS_β_**	AGACATGCTGTG	12
**^L^DNA_γ_**	p-TCTAAGCCAT*CCGCAAAAATGACCTCTTATC*AAAAGGAGCAA ttaaaggtactCTCTAATCCTGACCTGTTG	72
**^L^DNA_δ_**	p-GTCTGCTGCTATTTttggttctataGTTCTCCGTA*TACGTTTTCTTCAAA* *C*AGGTCTCC	59
**HS_I__′_**	*GTTTGAAGAAAACGTTAGATAAGAGGTCATTTTTGCGG*	38
**HS_II__′_**	agtacctttatgatagaaccaa	22
**RS_γ_**	CTTAGACAACAG	12
**RS_δ_**	GCAGACGGAGAC	12

Notes: The complementary parts between ^L^DNA_α_ and ^L^DNA_β_ are designated by dotted underlines. The HS_I_ sequence close to the 5′ terminus is complementary to a part of ^L^DNA_α_ (underlined italic), while the other part of HS_I_ is complementary to a part of ^L^DNA_β_ (italic). The HS_II_ sequence close to the 3′ terminus is complementary to ^L^DNA_α_ (underlined lowercase), while the other part of HS_II_ is complementary to ^L^DNA_β_ (lowercase). To ^L^DNA_α_, ^L^DNA_β_, ^L^DNA_γ_, and ^L^DNA_δ_, a terminal phosphate group, was attached for DNA ligation. The marks of the complementary regions for ^L^DNA_γ_, ^L^DNA_δ_, HS_I__′_, and HS_II__′_ systems are the same as above.
